# Correlation of Serum Levels of Endostatin with Tumor Stage in Gastric Cancer: A Systematic Review and Meta-Analysis

**DOI:** 10.1155/2015/623939

**Published:** 2015-01-19

**Authors:** Zheng-Hua Wang, Zhi-Tu Zhu, Xu-Yang Xiao, Jin Sun

**Affiliations:** ^1^Department of Tumor, The First Affiliated Hospital of Liaoning Medical University, Jinzhou 121001, China; ^2^Department of Thoracic Surgery, The First Affiliated Hospital of Liaoning Medical University, Jinzhou 121001, China

## Abstract

*Background*. We performed a systematic review and meta-analysis to study the association between serum endostatin levels and gastric cancer (GC) progression. *Method*. We searched the MEDLINE, Science Citation Index, Cochrane Library, PubMed, Embase, Current Contents Index, and several Chinese databases for published studies relevant to our study topic. Carefully selected studies were pooled and SMD and its corresponding 95% CI were calculated. Version 12.0 STATA software was used for statistical analysis. *Results*. Serum endostatin levels were analyzed in 12 case-control studies (736 GC patients and 350 controls). Significant differences in serum endostatin levels were observed between GC patients and the healthy controls (SMD = 1.418, 95% CI = 1.079~1.757, *P* < 0.001). Importantly, significantly lower levels of serum endostatin were found in I-II grade patients compared to those with III-IV grade tumors (*P* < 0.001). Further, higher serum endostatin levels were observed in the LN invasion-positive GC subjects in comparison with LN invasion-negative subjects (*P* < 0.001). *Conclusion*. Patients with GC exhibited elevated levels of serum endostatin than controls and its level showed a statistical correlation with the more aggressive type of GC, exhibiting invasion and LN metastasis. Thus, serum levels of endostatin being a useful prognostic biomarker for GC patients warrants further investigation.

## 1. Introduction

Gastric cancer (GC) is a malignant tumor arising from the stomach and is one of the most prevalent cancers in the world [1]. GC is ranked as the fourth most common cancer, causing more than 800,000 deaths worldwide each year. Despite the decline in mortality rates in recent decades, GC remains at the top, only second to lung cancer, in cancer related deaths [2, 3]. More than one million people are newly diagnosed with GC each year, imposing a heavy burden on world health services [4]. Both genetic and epigenetic risk factors contribute to the carcinogenesis and progression of GC [5, 6]. Factors such as gender, cigarette smoking, alcohol intake, diet, partial gastrectomy, and* Helicobacter pylori* infection are intimately linked to the pathogenesis of GC, and dietary conditions play the major role [6, 7]. Treatment approaches for GC vary widely depending on the tumor stage and characteristics at diagnosis, but treatment generally includes drugs and radical gastric excision [8]. For this reason, correct diagnosis of the disease is critical for making the right treatment decisions. There is, thus, a significant focus on identification of biomarkers for GC prevention and diagnosis [9]. In this context, endostatin is viewed with interest as a new biomarker for GC [10].

Endostatin was described as an endogenous tumor angiogenesis inhibitor and is a 183-amino acid proteolytic fragment produced from its precursor collagen XVIII [11]. Previous studies describe multiple roles for endostatin in modulating endothelial cell behavior; for example, endothelin induces endothelial cell apoptosis and acts as a regulator of tube formation and migration and growth of endothelial cells. Thus, endothelin interfered with tumor proliferation by inhibiting the activity of tumor-stimulating growth factors [12, 13]. In addition, a few studies have shown that endostatin inhibits tumor angiogenesis and tumor metastasis by limiting blood supply to tumors, thereby depriving tumors of nutrients, and was considered as a potential anticancer maker in treatment for malignant tumors [14–16]. However, higher concentrations of serum endostatin were soon found in various malignancies, such as breast cancer, non-small cell lung cancer, renal cell carcinoma, nasopharyngeal carcinoma, and soft tissue sarcoma [17, 18]. Besides, significantly elevated serum endostatin levels are found in GC patients [10, 19]. Higher serum endostatin level is seen in advanced GC and is associated with poor clinical outcome, with lower five-year survival rates [20, 21]. Therefore, it is reasonable to speculate that elevated serum endostatin levels can be used as a biomarker for early diagnosis and to predict the severity of GC [10]. Multiple studies demonstrated a close relationship between increased serum levels of endostatin and the tumor stage of GC [22, 23]. However, other studies have shown contradictory results [10, 24]. Therefore, we conducted the present meta-analysis to evaluate the potential value of endostatin as a diagnostic marker for the pathological features of GC and further investigated its role in assessing the grade of GC, as well as predicting the disease course.

## 2. Materials and Methods

This meta-analysis was conducted according to the guidelines of the preferred reporting items for systematic reviews and meta-analysis (PRISMA) statement on the quality of published systematic review and meta-analyses [25].

### 2.1. Search Strategy

Potential relevant studies were identified by a comprehensive literature search without language restriction, which covered the following computerized bibliographic databases: MEDLINE (1966~May 2014), Science Citation Index (1945~May 2014), Cochrane Library (Oxford, UK, Issue 12, 2014), PubMed (1966~May 2014), Embase (1974~May 2014), CINAHL (1982~May 2014), and Current Contents Index (1995~May 2014). Three Chinese databases (Chinese Biomedical, 1978~May 2014; the Chinese Journal Full-Text, 1980~May 2014; and Weipu Journal, 1989~May 2014) were also applied to identify Chinese-language articles. Studies published in Chinese and English were included in the present meta-analysis. We used the following medical subject headings and free language terms in conjunction with a highly sensitive search strategy: the search terms were (“stomach neoplasms” or “gastric cancer” or “stomach cancer” or “gastric neoplasms” or “gastric carcinomas” or “stomach carcinomas” or “carcinoma ventriculi”) and (“Endostatins” or “Endostatin”). Additionally, the references lists of relevant studies selected from the electronic debates were searched manually to find additional work.

### 2.2. Inclusion and Exclusion Criteria

To be included in the systematic review, retrieved studies were assessed for their suitability for meeting the following criteria: (1) the search results were conducted within a human population and published in a peer-reviewed journal; (2) only those case-control (healthy controls) or cohort studies examining the role of serum endostatin levels in tumorigenesis and progression of GC were incorporated into the meta-analysis; (3) cancer specimens were obtained from patients with histologically confirmed GC; (4) clinicopathological staging for each GC sample should be in accordance with the TNM system [26]; (5) the article must present original data and supply sufficient information on the serum endostatin levels in different clinicopathological features of GC; (5) the study should provide enough data to calculate an effect size; (6) once studies provided overlapping data, we would choose the study that had the largest number. The major exclusion criteria in this systematic review were as follows: (1) the articles that did not satisfy the current inclusion criteria; (2) some publication types, such as letters, abstracts, reviews, meta-analysis, and proceedings; (3) unpublished sources of data; (4) duplication publications. With the help of these inclusion criteria, the title and abstract of all the articles were evaluated on relevance. From the selected articles, the full texts were reviewed, followed by a decision on their eligibility for inclusion.

### 2.3. Study Quality and Data Extraction

Two experienced reviewers independently assessed the methodological quality of the included trials using the critical appraisal skills program (CASP, Milton Keynes Primary Care Trust, 2002, Institute of Health Sciences, Oxford) to ensure consistency in reviewing and reporting results (available at http://www.phru.nhs.uk/casp/qualitat.htm). And the specific contents of CASP were as follows: the study addresses a clearly focused issue (CASP01); the research problem is appropriate and the research design answers the research problem (CASP02); the cases are recruited in an acceptable way (CASP03); the controls are selected in an acceptable way (CASP04); the measurement for exposure factors is accurate to minimize bias (CASP05); the study controls other important confounding factors (CASP06); the research result is complete (CASP07); the research result is precise (CASP08); the research result is reliable (CASP09); the research result is applicable to the local population (CASP10); the research result fits with other available evidences (CASP11). Each of the two reviewers assessed the studies independently based on the inclusion/exclusion criteria. We used a standardized data form to collect the following descriptive information: surname and initials of the first author, the year of publication or submission, journal, source country, racial descent of study population, language of publication, study design, number of subjects, demographic variables of the subjects, detection methods of serum endostatin levels, clinicopathological characteristics, and so forth. Disagreement on the inclusion of a single study was settled by discussion, or a third investigator was consulted.

### 2.4. Statistical Analysis

The standardized mean differences (SMD) for the serum endostatin levels in GC patients and the controls were calculated, as well as the serum levels of endostatin in different clinicopathological stages. A 95% confidence interval (95% CI) was calculated for the summary SMD by the use of *Z* test. Also, a test for heterogeneity between trials included for each comparison was performed by the use of Cochran's *Q*-statistic and *I* ^2^ tests [27]. If the *Q*-test showed evidence of a *P* < 0.05 or *I* ^2^ test exhibited >50%, indicating maximal heterogeneity among the included studies, we performed metaregression analysis with a random-effects model to explore sources of heterogeneity, and otherwise SMDs were pooled in accordance with the fixed-effects model [28, 29]. When significant heterogeneity existed, the differences in endostatin levels (and 95% CI) were assessed for subgroups of different explanatory variables. Additionally, in order to evaluate the impact of single studies on the overall estimate, a one-way sensitivity analysis was employed. Further, Egger's linear regression test with visual inspection of the funnel plot and fail-safe number was applied to detect the potential publication bias [30, 31]. Statistical analyses were conducted with the STATA statistical software (Version 12.0, Stata Corporation, College Station, TX, USA).

## 3. Results

### 3.1. Description of Included Studies

**Figure 1:**
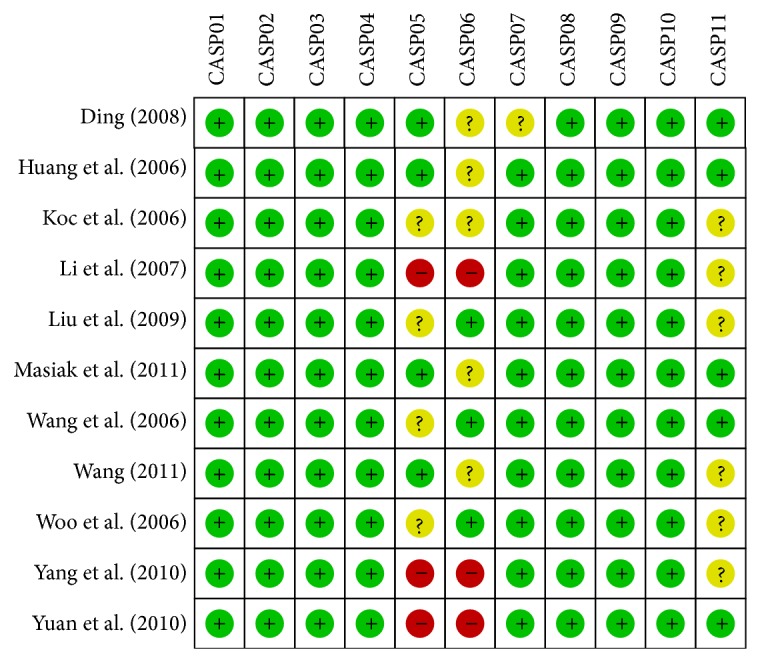
Quality assessment of included studies by CASP scores.

The combined electronic and manual search initially resulted in 135 potentially eligible articles. After the exclusion of 2 duplicate studies, the retrieved studies (*n* = 133) were screened by title and abstract for relevance, and subsequently, 73 irrelevant articles were excluded. Next, we systematically reviewed the remaining 60 articles for full-text reading. After full-text reading, 46 articles were deemed unsuitable and were therefore excluded, and 14 articles were selected for more detailed screening. After careful review, another 2 studies were excluded due to lack of data integrity. Finally, 12 clinical studies containing 1,086 subjects (736 GC patients and 350 controls) were incorporated into the current meta-analysis. The sample sizes in the 12 studies ranged from 30 to 130 participants [10, 22–24, 32–39]. All the enrolled studies showed moderate-high quality, as shown in Figure 1.

From the 12 included studies, 10 studies [10, 22, 23, 32–38] containing 922 subjects (sample size range, 30–130) measured the serum endostatin levels in GC patients and the controls, as well as in different clinicopathological stages, such as TNM stage, histologic grade, invasive grade, and LN metastasis. However, Li et al. did not contain data on the serum endostatin levels in different clinicopathological stages, and the controls in one study [39] were defined as the patients operated for benign pathologies, and thus the controls were not enrolled in our case-control study. One study was performed in Caucasians (Poland [10]) and the remaining 11 studies were in Asians (China [23, 24, 32–35, 37, 38], Korea [22], and Turkey [39]). Masiak et al. lacked information on both the gender and age in cases and the healthy controls; Ding did not provide gender and age information in controls. Detection of serum levels of endostatin was performed with ELISA and EIA [32, 39].  Table 1 shows the baseline characteristics and endostatin levels in cases/controls and the clinicopathological features of the individual studies.

### 3.2. Quantitative Data Synthesis

**Figure 2:**
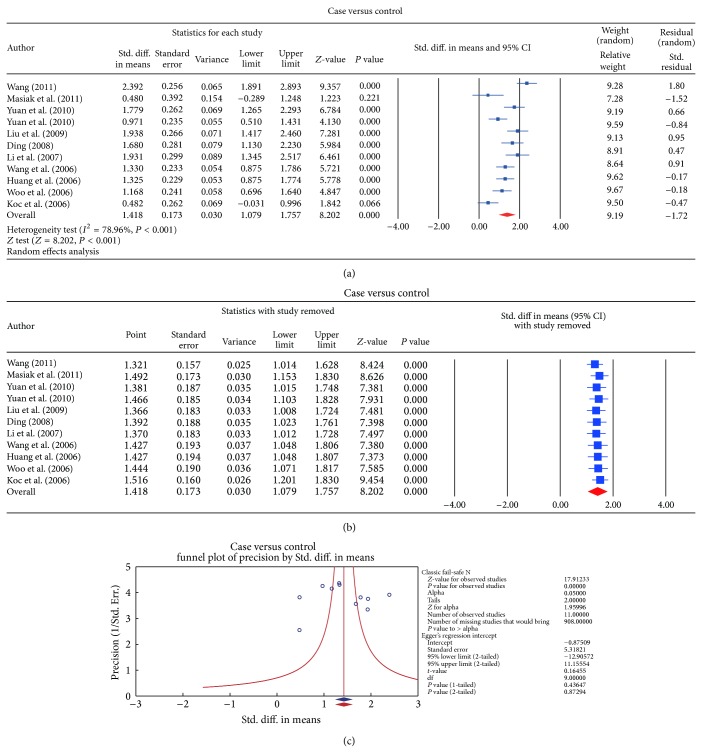
(a) Forest plots for the comparisons of serum endostatin levels between gastric cancer patients and healthy controls. (b) Sensitivity analyses for the comparisons of serum endostatin levels between gastric cancer patients and healthy controls. (c) Publication bias on the differences of serum endostatin levels between gastric cancer patients and healthy controls.

**Figure 3:**
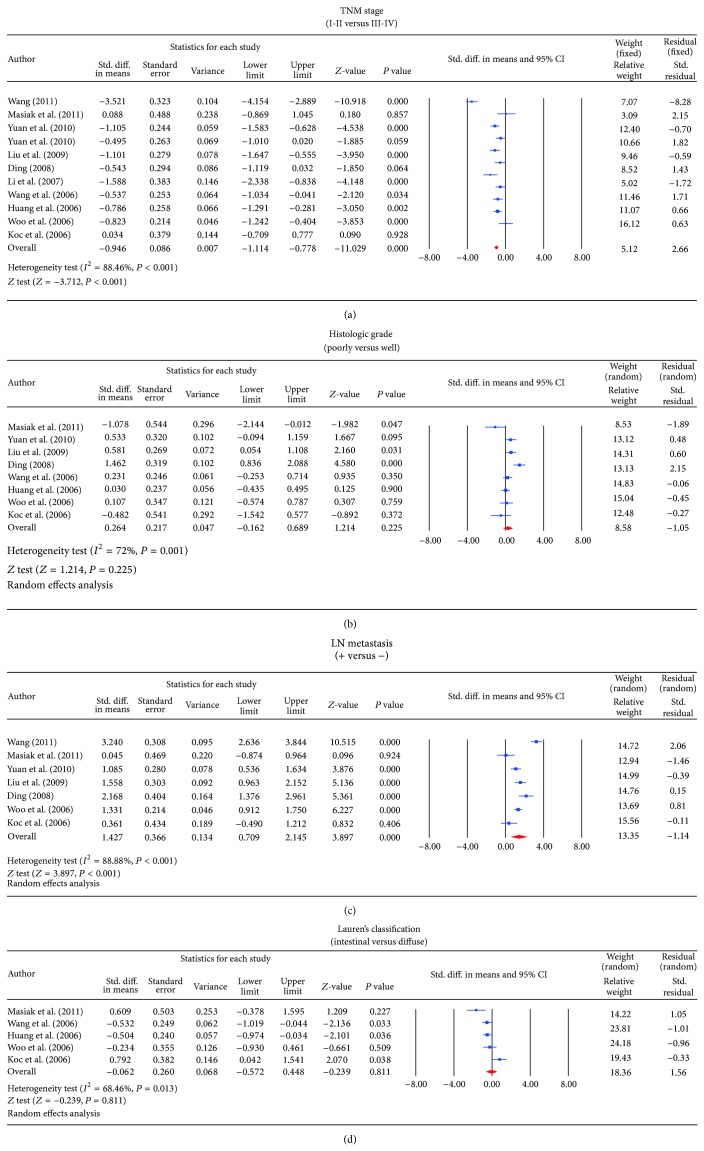
(a) Forest plots for the comparisons of serum endostatin levels in different stage of TNM in gastric cancer. (b) Forest plots for the comparisons of serum endostatin levels in the LN invasion-positive and LN invasion-negative gastric cancer. (c) Forest plots for the comparisons of serum endostatin levels between patients with poorly differentiated and well-differentiated gastric cancer. (d). Forest plots for the comparisons of serum endostatin levels between intestinal- and diffuse-type gastric cancers.

The following analyses were performed with a random-effects model for the evidence of *Q*-test and *I* ^2^ test (case versus controls: *I* ^2^ = 83.3%, *P* < 0.001; histological, T4 versus T2: *I* ^2^ = 95.9%, *P* < 0.001; LN metastasis, positive versus LN negative: *I* ^2^ = 88.8%, *P* < 0.001, resp.). In the meta-analysis, significant differences in serum levels of endostatin were observed between GC patients and control subjects, showing increased level of endostatin in GC patients according to the random effects pooled SMD in the 11 studies (SMD = 1.418, 95% CI = 1.079~1.757, *P* < 0.001) (Figure 2). When serum endostatin levels were considered in relation to different stage of TNM, the results of the present study revealed significantly lower serum endostatin level in I-II grade compared with the III-IV tumor grade (SMD = −0.946, 95% CI = −1.114~−0.778, *P* < 0.001). Further, significantly higher serum endostatin levels were observed in LN invasion-positive GC subjects compared to the lower levels seen in LN invasion-negative subjects (SMD = 1.427, 95% CI = 0.709~2.145, *P* < 0.001) (Figure 3). However, based on the histological grading for degree of differentiation, there was no statistically significant difference in the serum endostatin levels between patients with poorly differentiated and well-differentiated GC (SMD = 0.264, 95% CI = −0.162~0.689, *P* = 0.225). In addition, according to Lauren's classification, no apparent difference was also detected when comparing the serum levels of endostatin between intestinal- and diffuse-type GC patients (SMD = −0.062, 95% CI = −0.572~0.448, *P* = 0.811) (also shown in Figure 3).

**Figure 4:**
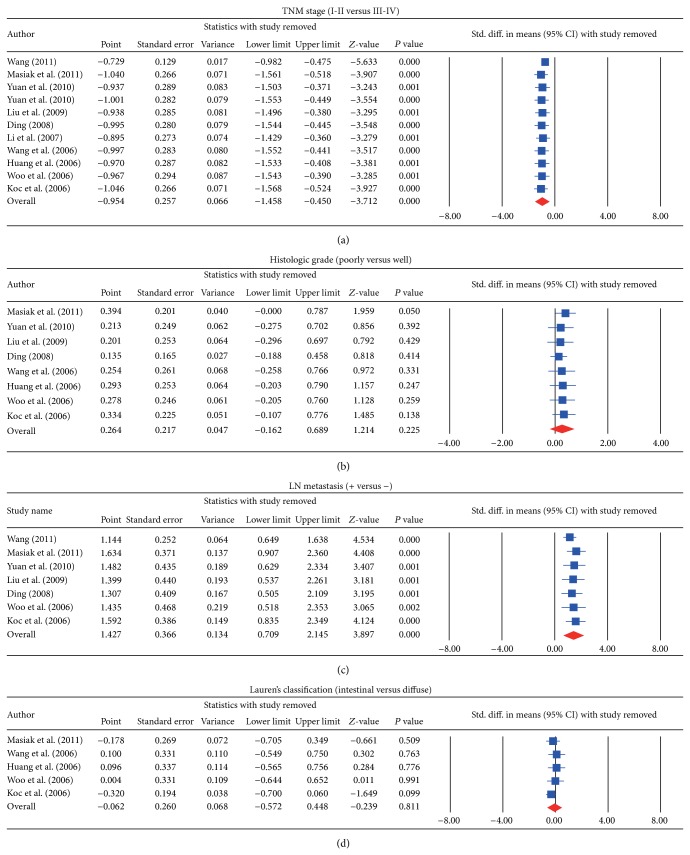
(a) Sensitivity analyses for the comparisons of serum endostatin levels in different stage of TNM in gastric cancer. (b) Sensitivity analyses for the comparisons of serum endostatin levels in the LN invasion-positive and LN invasion-negative gastric cancer. (c) Sensitivity analyses for the comparisons of serum endostatin levels between patients with poorly differentiated and well-differentiated gastric cancer. (d) Sensitivity analyses for the comparisons of serum endostatin levels between intestinal- and diffuse-type gastric cancers.

**Figure 5:**
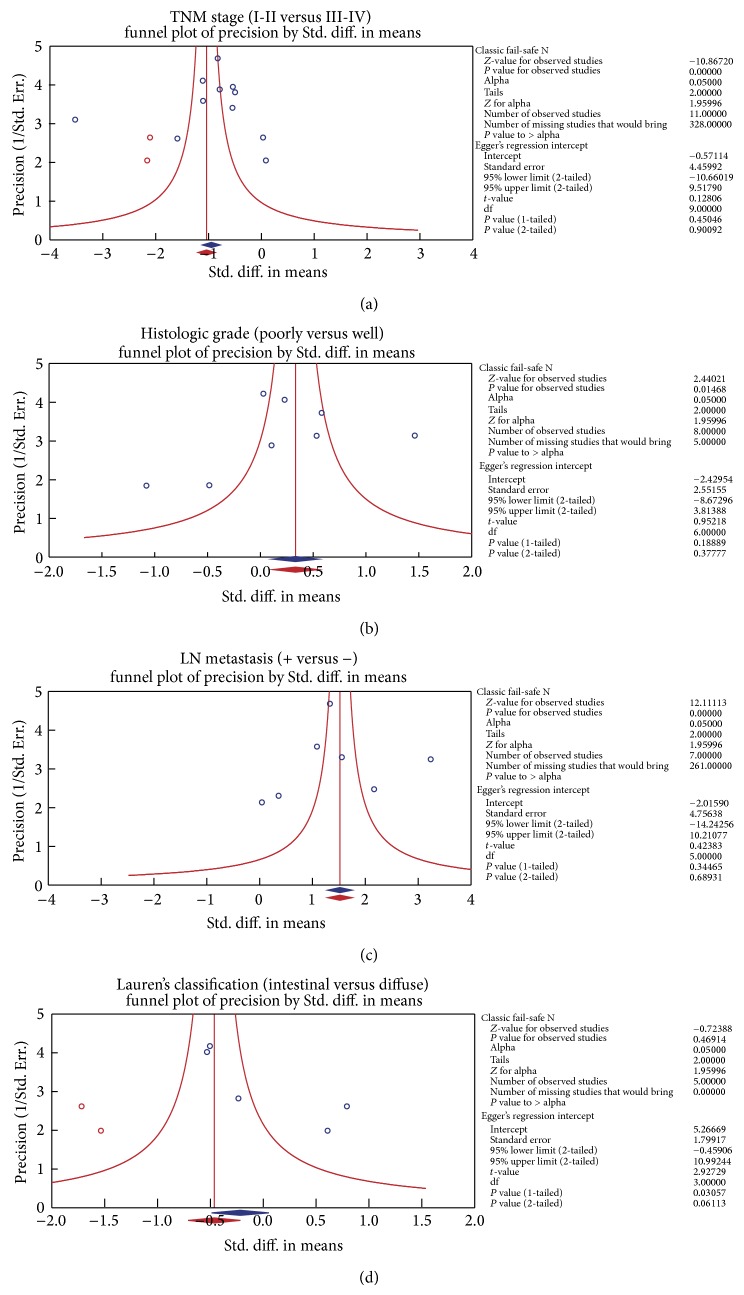
(a) Publication bias on the differences of serum endostatin levels in different stage of TNM in gastric cancer. (b) Publication bias on the differences of serum endostatin levels in the LN invasion-positive and LN invasion-negative gastric cancer. (c) Publication bias on the differences of serum endostatin levels between patients with poorly differentiated and well-differentiated gastric cancer. (d) Publication bias on the differences of serum endostatin levels between intestinal- and diffuse-type gastric cancers.

**Figure 6:**
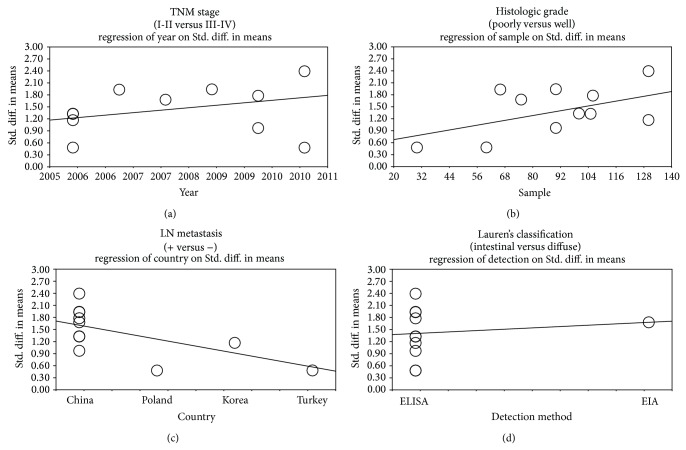
Univariate and multivariate regression analyses of potential source of heterogeneity: (a) year of publication, (b) sample size, (c) country, and (d) detection method.

We further conducted sensitivity analyses to determine whether review conclusions were affected by the choice of a single study; the finding suggested that no single study had the effect on the pooled SMDs in this meta-analysis (Figures 2 and 4). Finally, the forest plot resembled a symmetrical, inverted funnel, suggesting absence of bias (Figures 2 and 5). Egger's regression test also showed no evidence of asymmetrical distribution (all *P* > 0.05) in the systematic reviews; higher fail-safe number meant better reliability of the meta-analysis. As shown in Figure 6 and Table 2, univariate and multivariate regression analysis indicated that the year of publication, sample size, country, and detection method were not the major source of heterogeneity in the present meta-analysis and might not be the key factors affecting the overall effects (all *P* > 0.05).

## 4. Discussion

Our meta-analysis integrated evidence from multiple relevant studies in order to assess the connection between serum levels of endostatin and pathological characteristics of GC to gain an insight into the role of endostatin in the development of GC. The major results of our statistical analysis revealed that serum endostatin levels in GC patients were higher than those of healthy subjects, implying that serum levels of endostatin might reflect the pathogenesis of GC. Endostatin was described as an antigrowth factor and antiendothelial cell migration factor, inhibiting angiogenesis by reducing the blood supply necessary for tumor growth [10, 40]. Since endostatin can be secreted by both normal endothelium cells and tumor cells, increased serum levels of endostatin in GC patients can be explained by the hypothesis that endostatin is generated by a negative feedback mechanism in an attempt to repress or offset the upregulated angiogenesis in tumors [39].

Furthermore, our findings showed that patients with stage T4 GC had higher serum levels of endostatin than those with stage T2 GC, suggesting that serum endostatin levels might be related to the aggressiveness of GC. Endostatin has various antitumor functions through regulating a variety of receptors, including inhibition of angiogenesis and repression of migration and invasion of tumor cells [41, 42]. Consequently, it is plausible that increased serum endostatin levels in advanced (stage T4) GC may be ascribed to production by cancer tissues or to a host response to rectify the imbalance of abnormal angiogenic stimuli during the progression of tumors [20, 43]. On the other hand, a recent study presented strong arguments that the endothelin is a potent mediator of systemic inflammation and this could better explain its correlation with advanced tumor stages. The results of the present meta-analysis also indicated that serum endostatin levels of patients with LN metastasis were increased compared with those of patients without LN metastasis, indicating that serum endostatin levels may be involved in the progression of GC. Consistent with our findings, Fujita et al. also found that serum levels of endostatin are upregulated in GC patients, showing that serum endostatin levels could be an important prognostic biomarker in predicting the survival of patients with metastatic GC [20].

We also performed stratified analysis on the basis of country and detection method so as to obtain a better understanding of serum endostatin levels in the development of GC. In the subgroup analysis by country, we observed that GC patients exhibited higher serum endostatin level among Chinese and Koreans but not among Polish population. Furthermore, serum endostatin levels were associated with histological stages of GC only among the Chinese. Our results also demonstrated a strong positive correlation between serum endostatin levels and LN metastasis in GC among the Chinese and Koreans, while such relationship was not found among Polish and Turkish populations. In short, the discovery of the current meta-analysis was in conformity with previous studies that serum endostatin levels may be helpful in detection of GC and may be also utilized to predict the progression of GC.

Meanwhile, there are limitations to the current meta-analysis that should be noted. First, there is the possible existence of biases; although we performed a methodological assessment to avoid selection biases, there was significant heterogeneity among the 12 articles which may be attributed to the nonuniform techniques of detecting endostatin. Overall estimate of SMD was only suggestive given the highly significant heterogeneity across studies. Second, a potential publication bias existed in this study, since we did not take several unpublished articles and abstracts into account due to unavailable data. In addition, language may also introduce bias, especially since we selected studies published only in English or Chinese studies and ignored all other languages. A third potential limitation is its relative small sample size; therefore, results of this study will need to be confirmed in prospective randomized controlled trials with larger study population. Despite the above limitations, this is the first example of meta-analysis on the association of serum endostatin levels with the development of GC.

Taken together, our results indicate that the increased serum level of endostatin may contribute to an aggressive LN metastasis, either through activation of multiple signaling pathways or through producing robust systemic inflammatory responses, to promote aggressive invasive behavior of GC. Serum levels of endostatin may help clinicians to make difficult therapeutic decisions for GC. However, based on the above limitations, further in-depth study is recommended in confirming the exact role of serum endostatin levels for GC progression.

## Figures and Tables

**Table 1:** Characteristics of included studies focused on protein expression of endostatin.

First author	Year	Country	Sample size		Gender (M/F)		Age (years)		Method	CASP
			Case	Control	Case	Control	Case	Control		
Wang [36]	2011	China	100	30	68/32	20/10	57.2 ± 12.4	57.2 ± 12.4	ELISA	8
Masiak [10]	2011	Poland	20	10	—	—	—	—	ELISA	5
Yuan [38]	2010	China	82	24	56/26	16/8	59 (27~84)	59 (27~84)	ELISA	8
Yang [37]	2010	China	60	30	34/26	28/12	38~76	32~68	ELISA	7
Liu [34]	2009	China	60	30	37/23	17/13	53.3 ± 8.2	38.3 ± 7.8	ELISA	7
Li [24]	2009	China	52	52	33/19	33/19	62 (38~75)	62 (38~75)	ELISA	6
Ding [32]	2008	China	50	25	30/20	—	62 (30~85)	—	EIA	6
Li [33]	2007	China	36	30	23/13	19/11	58.7 ± 7.9	58.7 ± 7.9	ELISA	6
Wang [35]	2006	China	67	33	37/30	19/14	48 (35~73)	46 (31~69)	ELISA	7
Huang [23]	2006	China	72	33	38/34	19/14	48 (35~73)	46 (31~69)	ELISA	8
Woo [22]	2006	Korea	107	23	70/37	15/8	60 (31~78)	58 (34~75)	ELISA	8
Koç [39]	2006	Turkey	30	30	10/20	11/19	41.3 ± 16.7	64.3 ± 16.7	EIA	6

M: male; F: female; ELISA: enzyme-linked immunosorbent assay; CASP: critical appraisal skills program.

**Table 2:** Metaregression analyses of potential source of heterogeneity.

Heterogeneity factors	Coefficient	SE	*t*	*P*	95% CI	
				(Adjusted)	LL	UL
Year	0.033	0.081	0.42	0.985	−0.164	0.232
Sample	0.009	0.006	1.66	0.386	−0.004	0.023
Country	−0.283	0.167	−1.69	0.365	−0.691	0.126
Detecting method	0.288	0.533	0.54	0.969	−1.016	1.593

*Notes.* SE: standard error; LL: lower limit; UL: upper limit.

## References

[1] Yasui W., Sentani K., Sakamoto N., Anami K., Naito Y., Oue N. (2011). Molecular pathology of gastric cancer: research and practice. *Pathology Research and Practice*.

[2] Singh S. R. (2013). Gastric cancer stem cells: a novel therapeutic target. *Cancer Letters*.

[3] Ferro A., Peleteiro B., Malvezzi M. (2014). Worldwide trends in gastric cancer mortality (1980–2011), with predictions to 2015, and incidence by subtype. *European Journal of Cancer*.

[4] Dominguez R. L., Crockett S. D., Lund J. L. (2013). Gastric cancer incidence estimation in a resource-limited nation: use of endoscopy registry methodology. *Cancer Causes and Control*.

[5] Lee Y.-S., Cho Y. S., Lee G. K. (2014). Genomic profile analysis of diffuse-type gastric cancers. *Genome Biology*.

[6] Kim J., Cho Y. A., Choi I. J. (2013). Effects of polymorphisms of innate immunity genes and environmental factors on the risk of noncardia gastric cancer. *Cancer Research and Treatment*.

[7] Montori G., Coccolini F., Ceresoli M. (2014). The treatment of peritoneal carcinomatosis in advanced gastric cancer: state of the art. *International Journal of Surgical Oncology*.

[8] Arkenau H.-T. (2009). Gastric cancer in the era of molecularly targeted agents: current drug development strategies. *Journal of Cancer Research and Clinical Oncology*.

[9] Nie X.-C., Wang J.-P., Zhu W. (2013). COL4A3 expression correlates with pathogenesis, pathologic behaviors, and prognosis of gastric carcinomas. *Human Pathology*.

[10] Masiak W., Szponar A., Chodorowska G., Da̧browski A., Pedowski T., Wallner G. (2011). Evaluation of endostatin and EGF serum levels in patients with gastric cancer. *Polish Journal of Surgery*.

[11] Zhuo W., Chen Y., Song X., Luo Y. (2011). Endostatin specifically targets both tumor blood vessels and lymphatic vessels. *Frontiers of Medicine in China*.

[12] Jiang W.-G., Lu X.-A., Shang B.-Y. (2013). Genetically engineered endostatin-lidamycin fusion proteins effectively inhibit tumor growth and metastasis. *BMC Cancer*.

[13] Wan Y.-Y., Tian G.-Y., Guo H.-S. (2013). Endostatin, an angiogenesis inhibitor, ameliorates bleomycin-induced pulmonary fibrosis in rats. *Respiratory Research*.

[14] Zhuo W., Luo C., Wang X., Song X., Fu Y., Luo Y. (2010). Endostatin inhibits tumour lymphangiogenesis and lymphatic metastasis via cell surface nucleolin on lymphangiogenic endothelial cells. *The Journal of Pathology*.

[15] Dong X., Zhao X., Xiao T., Tian H., Yun C. (2011). Endostar, a recombined humanized endostatin, inhibits lymphangiogenesis and lymphatic metastasis of Lewis lung carcinoma xenograft in Mice. *Thoracic and Cardiovascular Surgeon*.

[16] Bai Y.-J., Huang L.-Z., Zhou A.-Y., Zhao M., Yu W.-Z., Li X.-X. (2013). Antiangiogenesis effects of endostatin in retinal neovascularization. *Journal of Ocular Pharmacology and Therapeutics*.

[17] Mo H.-Y., Luo D.-H., Qiu H.-Z. (2013). Elevated serum endostatin levels are associated with poor survival in patients with advanced-stage nasopharyngeal carcinoma. *Clinical Oncology*.

[18] Ni Q., Ji H., Zhao Z., Fan X., Xu C. (2009). Endostar, a modified endostatin inhibits non small cell lung cancer cell *in vitro* invasion through osteopontin-related mechanism. *European Journal of Pharmacology*.

[19] Li L.-X., Zhang Y.-L., Zhou L. (2013). Antitumor efficacy of a recombinant adenovirus encoding endostatin combined with an E1B55KD-deficient adenovirus in gastric cancer cells. *Journal of Translational Medicine*.

[20] Fujita T., Gotohda N., Kato Y. (2012). Clinicopathological features of stomach cancer with invasive micropapillary component. *Gastric Cancer*.

[21] Lurje G., Husain H., Power D. G. (2009). Genetic variations in angiogenesis pathway genes associated with clinical outcome in localized gastric adenocarcinoma. *Annals of Oncology*.

[22] Woo I. S., Kim K.-A., Jeon H.-M. (2006). Pretreatment serum endostatin as a prognostic indicator in metastatic gastric carcinoma. *International Journal of Cancer*.

[23] Huang X. F., Zhou J. B., Du G., Pan X. H., Xu R. H. (2006). Clinical significace of serum VEGF and endostatin combined detection in patients with gastric cancer. *Chinese Journal of Digestive Endoscopy*.

[24] Li G., Li L. H., Song Y. G. (2009). Expression of TP and ES in the patients with gastric cancer and its clinical significance. *Jilin Medical Journal*.

[25] Panic N., Leoncini E., de Belvis G., Ricciardi W., Boccia S. (2013). Evaluation of the endorsement of the preferred reporting items for systematic reviews and meta-analysis (PRISMA) statement on the quality of published systematic review and meta-analyses. *PLoS ONE*.

[26] Edge S. B., Compton C. C. (2010). The american joint committee on cancer: the 7th edition of the AJCC cancer staging manual and the future of TNM. *Annals of Surgical Oncology*.

[27] Zintzaras E., Ioannidis J. P. A. (2005). HEGESMA: genome search meta-analysis and heterogeneity testing. *Bioinformatics*.

[28] Zintzaras E., Ioannidis J. P. A. (2005). Heterogeneity testing in meta-analysis of genome searches. *Genetic Epidemiology*.

[29] Higgins J. P. T., Thompson S. G. (2002). Quantifying heterogeneity in a meta-analysis. *Statistics in Medicine*.

[30] Song F., Gilbody S. (1998). Bias in meta-analysis detected by a simple, graphical test. Increase in studies of publication bias coincided with increasing use of meta-analysis. *British Medical Journal*.

[31] Peters J. L., Sutton A. J., Jones D. R., Abrams K. R., Rushton L. (2006). Comparison of two methods to detect publication bias in meta-analysis. *Journal of the American Medical Association*.

[32] Ding H. H. (2008). Clinical significace of serum VEGF and endostatin combined with pathological features in patients with gastric cancer. *Zhejiang Clinical Medical Journal*.

[33] Li Z., Zhang H. Y., Zai S. F., Li B. D., Xie Z. B., Yue A. M. (2007). Level and significance of vascular endothelial growth factor and endothelium colyone of gastric carcinoma. *Chinese Journal of Postgraduates of Medicine*.

[34] Liu F. G., Zhoiu H. M., Xu J. M., Luan C. Y., Zhang Y. H., Zhao C. X. (2009). Relationships between serum levels of vascular endothelial growth factor and endostatin in patients with gastric carcinoma and clinical pathological characteristics. *Shandong Medical Journal*.

[35] Wang S., Huang X., Xu X. H., Chen W. H. (2006). Relation of serum endostatin levels with clinicopathologic characteristics and prognosis of gastric carcinoma. *Journal of Practical Oncology*.

[36] Wang S. D. (2011). Clinical significace of serum MMP-9, VEGF and endostatin combined detection in patients with gastric cancer. *Shandong Medical Journal*.

[37] Yang Y. E., Meng Q. S., Sun X. J. (2010). Clinical significace of serum CEACA724VEGF and endostatin combined detection in patients with gastric cancer. *Medical Laboratory Science and Clinics*.

[38] Yuan H. Z., Lv C., Huang X. W., He C. (2010). Serum levels of vascular endothelial growth factor and endostatin in patients with gastric cancer and their clinical significance. *The Journal of Practical Medicine*.

[39] Koç M., Göçmen E., Kiliç M., Özbay M., Öktem M., Tez M. (2006). Serum endostatin levels in gastric cancer patients: correlation with clinicopathological parameters. *Hepato-Gastroenterology*.

[40] Ren B., Höti N., Rabasseda X., Wang Y.-Z., Wu M. (2003). The antiangiogenic and therapeutic implications of endostatin. *Methods and Findings in Experimental and Clinical Pharmacology*.

[41] Folkman J. (2006). Antiangiogenesis in cancer therapy—endostatin and its mechanisms of action. *Experimental Cell Research*.

[42] Xu R., Ma N., Wang F. (2013). Results of a randomized and controlled clinical trial evaluating the efficacy and safety of combination therapy with Endostar and S-1 combined with oxaliplatin in advanced gastric cancer. *OncoTargets and Therapy*.

[43] Hu T.-H., Huang C.-C., Wu C.-L. (2005). Increased endostatin/collagen XVIII expression correlates with elevated VEGF level and poor prognosis in hepatocellular carcinoma. *Modern Pathology*.

